# The effect of tropical cyclone on cognitive function in older adults: a longitudinal study from Thailand

**DOI:** 10.1093/gerona/glaf169

**Published:** 2025-07-29

**Authors:** Jin Ke, Fei Sun, Phatchanun Vivarakanon

**Affiliations:** Aging Service Research Center, School of Sociology, Huazhong University of Science and Technology, Wuhan, China; School of Social Work, Michigan State University, East Lansing, MI, United States; Boromarajonani College of Nursing, Nakhon Lampang, Faculty of Nursing, Praboromarajchanok Institute, Thailand

**Keywords:** Tropical cyclone, Calculation, Cognition, Long-term effect, Thailand, Climate change

## Abstract

**Background:**

In the context of climate change, tropical cyclones (TCs) pose an escalating threat to human health. This study examined the effects of TCs on cognitive function of older adults in Thailand and explored underlying mechanisms.

**Methods:**

Data came from 2 sources: the Health, Aging and Retirement in Thailand (HART) survey and the Emergency Events Database (EM-DAT). The 2 datasets were matched to estimate the short- and long-term effects of TC exposure on cognition using a fixed-effects model. Cognition of older adults was assessed along 3 dimensions: memory, calculation, and time orientation. Depression, hypertension, and social isolation were examined as potential underlying mechanisms.

**Results:**

Analyses found that exposure to TCs had a persistent negative effect on the calculation dimension, while its effects on memory and time orientation were minimal or short-lived. Specifically, exposed individuals had significantly lower calculation scores compared to unexposed individuals on the day of exposure, and this negative effect persisted for up to 4 years. An increased likelihood of depression, exacerbation of existing hypertension, and reduced engagement in social activities were found to help explain the effects of TCs on cognition.

**Conclusions:**

The study provided evidence of the detrimental effects of TCs on specific cognitive domains in older adults, identifying depression, exacerbation of hypertension, and social isolation as underlying mechanisms. The findings underscored the need for further research on the cognitive impact of TCs in the aging population, and the development of practice and policy interventions to mitigate these effects.

## Introduction

Tropical cyclones (TCs), such as tropical storms, typhoons, and hurricanes, have a devastating impact on human health due to their immense destructive power.^1^ An estimated 97 430 excess deaths occur per decade among the global population exposed to TCs for 2 weeks.^2^ Residents of East and South Asia are the most affected, with 28 744 and 27 267 excess deaths per decade, respectively.^2^ As global climate change progresses, the average frequency and intensity of TCs continue to rise, potentially exacerbating their negative impacts.^3^^,^^4^ This highlights a need to expand our understanding of the health consequences of TCs.

Current literature on the health impacts of TCs has identified various adverse outcomes, such as posttraumatic stress disorder (PTSD), chronic illness, and increased hospitalization rates and even mortality. However, less attention has been paid to their impact on cognitive function.^5^^,^^6^ Meanwhile, literature on cognitive function promotion^7^ has increasingly identified environmental hazards, such as air pollution and extreme temperature associated with climate change, as risk factors. Even subtle impacts on cognitive function in older adults can increase the likelihood for future development of dementia.^8^ Dementia has become a major global public health crisis, with 75.63 million people globally expected to live with dementia by 2030, at a total cost of $2 trillion.^9^ Given the limited effectiveness and high cost of dementia drug therapy, early detection and prevention or intervention strategies for cognitive decline in older adults are crucial.^10^ This underscores the need to identify any modifiable risk factors, including recognizing and then addressing natural disasters (eg, TCs) as risk factors for cognitive function in the context of climate change.^11^

Furthermore, existing studies have focused on assessing the health impacts of individual extreme TC events, mostly using a case-study approach limited by small sample size and cross-sectional design, with insufficient attention paid to the long-term impacts of TCs.^6^^,^^12^^,^^13^ Additionally, there remains a gap in understanding the impacts of TCs among the most vulnerable groups, such as older adults, which is critical to improving disaster preparedness.^14^ This study aims to fill this gap by examining the short- and long-term effects of TCs on cognitive function in older adults.

We conceptualize that TCs can have direct and indirect effects on the cognitive function of older adults. The direct negative effect of TCs on cognitive function may be explained by neuroscientific mechanisms, such as TC-induced acute and chronic stress, which can decrease cingulate white matter density in the short and long term after a disaster, leading to a decline in cognitive function.^15–17^ TCs can also lead to cognitive function decline through indirect mechanisms. Numerous studies have established the links between exposure to TCs and depression,^18–20^ hypertension,^21–23^ and social isolation.^24–26^ For example, Harville et al. found that postpartum women who experienced Hurricane Katrina had a significantly increased risk of depression.^18^ Similarly, World Trade Center rescue workers who endured Hurricane Sandy exhibited a higher risk of major depressive disorder,^19^ with the same adverse effect observed in older adults exposed to Hurricane Sandy.^20^ In terms of hypertension, studies of Hurricane Sandy and Typhoon Morakot confirmed a high prevalence of hypertension in areas severely affected by TCs^21^^,^^22^ along with a significantly higher risk of deterioration among those already living with hypertension.^23^ Additionally, many studies have shown that natural disasters may cause social isolation and limited access to social support.^24–26^ All 3 factors are recognized as significant predictors of cognitive function decline.^27–29^ Therefore, we speculate that these factors may be indirect pathways linking TCs to cognitive function decline in older adults.

Nonetheless, existing studies primarily assessed the impact of a single large cyclone on these factors, constrained by small sample sizes and cross-sectional data limiting generalizability across all TCs.^30^ Furthermore, few have examined the potential indirect mechanisms of TCs and focused on the aging population. Hence, this study aimed to illuminate the effect of TCs on cognitive function in older adults in Thailand and to explore potential underlying mechanisms.

Thailand was chosen for this focused study because it faces the dual challenges of aging and climate change as a developing country in Southeast Asia. In 2023, Thailand had a population of approximately 71.8 million, with about 20.9% aged 60 or older and 16.0% aged 65 or above.^31^ By 2050, these proportions are projected to increase to 38.3% and 31.6%, respectively. Among those aged 65 and older, around 26.4% remain in the workforce, highlighting the importance of their health.^31^

Thailand faces significant climate challenges due to its geographic location near the equator and its tropical climate, making it particularly vulnerable to TCs. TCs affecting Thailand approach from 2 directions: from the Bay of Bengal toward the western part of the country in May, and from the Pacific Ocean toward the eastern part from June to December, with September and October being the peak months for TCs.^32^ The occurrence of TCs overlaps with the Southwest Monsoon season, which originates from high-pressure areas in the Southern Hemisphere over the Indian Ocean and affects Thailand from mid-May to mid-October.^32^ These climatic conditions pose considerable challenges for older Thai adults.

## Methods

This study utilized data from 2 secondary sources: the Health, Aging, and Retirement in Thailand (HART) survey for variables of the older participants, and the Emergency Events Database (EM-DAT) for TC records.

### Study population

**Figure 1.**
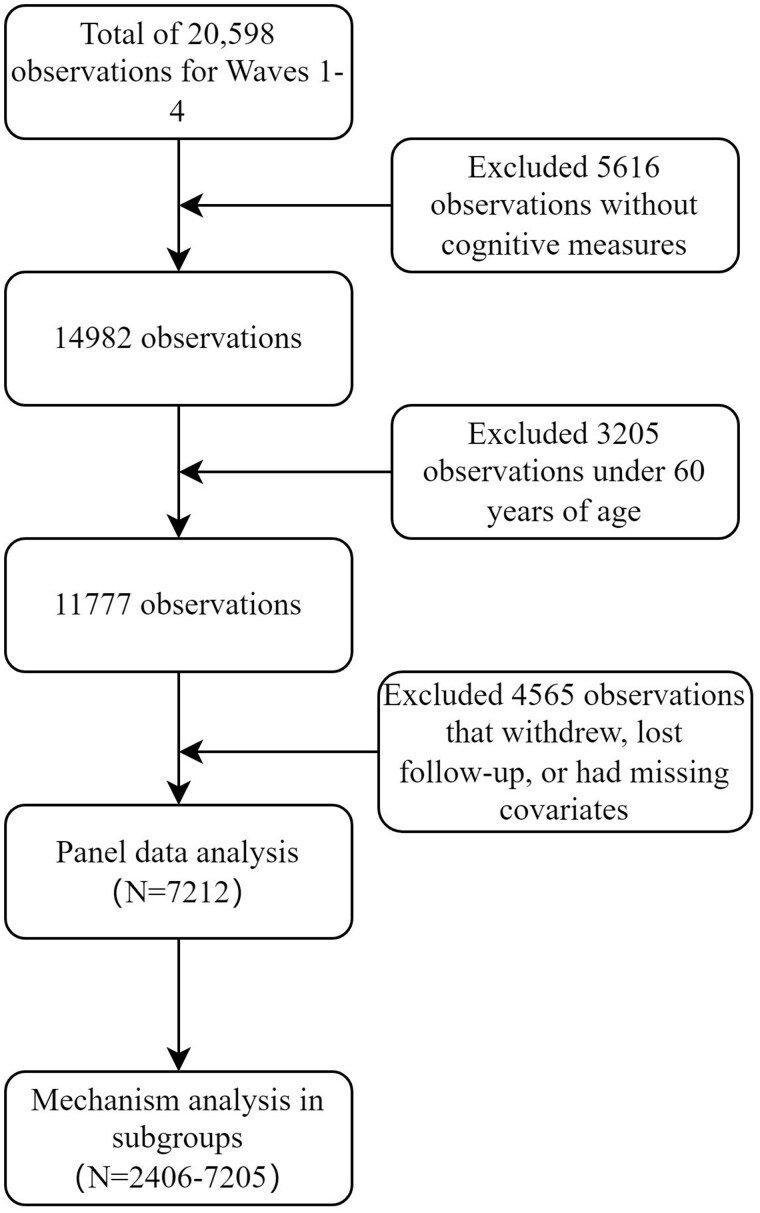
Flowchart of sample selection.

The HART survey, a sister study to the Health and Retirement Study in the United States, is based on a representative sample of approximately 5600 randomly selected households stratified across 5 regions of the country, including Bangkok and its surrounding areas. In each household, 1 individual aged 45 years or older was selected as the primary respondent. The survey’s primary objective was to create a nationwide longitudinal household panel dataset that captures a range of demographic, personal, and household characteristics, including health and cognition, employment and retirement, income, and life expectancy and life satisfaction.^33^ To date, HART has released 4 waves of data: 2015 (Wave 1), 2017 (Wave 2), 2020 (Wave 3), and 2022 (Wave 4), totaling 20 598 observations. As cognition was not assessed in Wave 1, this study only included data from Waves 2 through 4 that had measures of cognitive function. Furthermore, we excluded 3205 observations under the age of 60 as this study focused on older adults, defined in Thailand as people aged 60 or above.^34^ This study further excluded observations due to withdrawal, loss to follow-up, or missing covariates, leaving 7212 observations for final analysis. For the 3 mechanism analyses, we used the listwise deletion method, with a sample size ranging from 2406 to 7205. The sample selection process is shown in Figure 1.

### TC exposure

The TC records for this study were obtained from the EM-DAT maintained by the Center for Research on the Epidemiology of Disasters at the University of Leuven. EM-DAT is an international disaster database that includes global disasters that meet at least 1 of the following criteria: at least 10 deaths, 100 or more people affected, declaration of a national emergency, or provision of international assistance, and details of these disasters are recorded.^35^ EM-DAT database has been widely used in the field of public health and medicine.^35^^,^^36^

**Figure 2.**
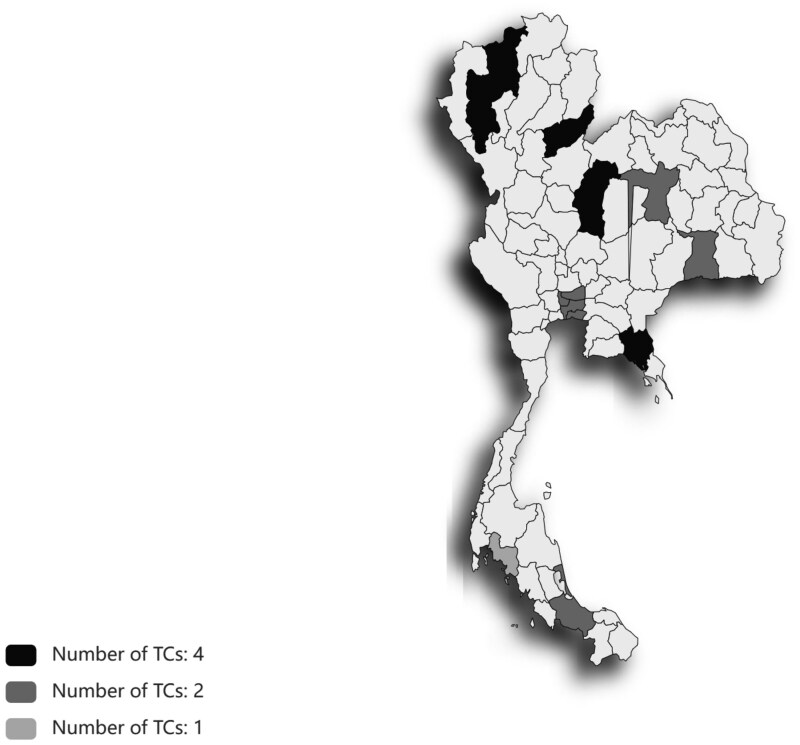
The distribution of TCs in the 13 provinces included in the 2017-2022 HART sample. HART, Health, Aging and Retirement in Thailand; TCs, tropical cyclones.

Data of TCs in Thailand from the EM-DAT database from 2017 to 2022, corresponding to the time frame of the sample set in the HART survey, were retrieved. Figure 2 shows the distribution of TCs in the 13 provinces included in the 2017-2022 HART sample. Detailed information is provided in Supplementary Table 1. We merged TC records with HART data at the provincial level across 3 HART waves in this study. In line with related studies,^6^^,^^37^^,^^38^ this study defined TC exposure by time of exposure using 7 variables: (1) Same-day exposure, defined as TC experiences that occurred on the same day as the date of the personal interview. (2) Exposure within 1 week, defined as TC experiences that occurred within 1 to 7 days before the interview date. (3) Exposure within 1 month, defined as TC experiences between 1 week and 1 month before the interview date. (4) Exposure within 3 months, defined as TC experiences that occurred within 1 month to 3 months before the interview date. (5) Exposure within one year, defined as TC experiences from 3 months to one year prior to the interview date. (6) Exposure within 4 years, defined as TC experiences that occurred from 1 year to 4 years prior to the interview date. (7) Exposure over 4 years, defined as experiences with TCs that occurred more than 4 years before the interview date. All 7 exposure variables were coded as dummy variables to avoid double counting of TC exposure experiences. Each variable had a value of 1 if the individual was exposed to TCs in the corresponding time before the interview date and 0 otherwise.

### Cognitive function

Cognitive function was assessed along 3 dimensions: (1) memory, measured by word recall tests, using immediate and delayed recall tests, each containing 10 words for a total of 20 points; (2) calculation, measured by the serial-7s test, which required individuals to respond to consecutive subtractions of 7 from 100 for a total of 5 times, with one point scored for each correct answer for a total of 5 points; and (3) time orientation, measured by awareness of time, requiring individuals to respond to the year, the month, the day, and the day of the week of the interview date, with one point scored for each correct answer for a total of 4 points.^39^ Descriptive statistics of the cognitive test scores are detailed in Supplementary Table 2.

### Potential indirect mechanisms

Three proposed underlying mechanisms included depression, hypertension, and social isolation. Depression was measured by the 10-item version of the Center for Epidemiologic Studies Depression Scale (CESD-10). Individuals with a CESD score of 10 or greater were considered to have depressive symptoms and were assigned a value of 1, otherwise were assigned 0. Specific procedures for CESD-10 scoring can be found elsewhere.^40^ Hypertension was scored in 2 ways: presence (1 for having hypertension, 0 for not) and severity for those with hypertension (1 for more severe, 0 for less severe). Social isolation was measured by participation in social activities (1 for nonparticipation, 0 for participation). Descriptive statistics of potential indirect mechanism variables are detailed in Supplementary Table 3.

### Covariates

Covariates included individual, household, and provincial factors. Individual-level covariates encompassed demographic and health-related characteristics, such as age, sex, marital status, years of education, employment status, religion, living arrangement, living in an urban or rural area, smoking status, alcohol consumption, exercise habits, and utilization of medical resources. Residents living in municipal districts are urban residents, and municipal districts include large cities, medium-sized cities, and township municipalities. Nonmunicipal areas are rural areas with lower population density and lack of urbanized services. Those who had previously smoked or drank and are still smoking or drinking are defined as smokers or drinkers, and those who have never smoked or drank or have previously smoked or drank and are now abstinent are defined as nonsmokers or nondrinkers. Individuals who would not exercise were considered to have no exercise habits, and individuals who exercised 1-7 days per week were considered to have exercise habits. The utilization of medical resources was measured by whether or not individuals used outpatient services, which were categorized as using hospital outpatient care, community health center outpatient care, and clinic outpatient care. Household-level covariates include the logarithm of household assets. Household assets include the current value of the house, the balance of deposits in all bank accounts, the value of capital held in companies or invested in the capital market, and the total value of motor vehicles. Furthermore, this study controlled for 2 provincial-level covariates: gross domestic product (GDP) per capita, obtained from the National Economic and Social Development Office of Thailand, and vegetation cover, measured by the normalized difference vegetation index (NDVI) provided by the Food and Agriculture Organization of the United Nations. These are considered as potential confounders.^41^^,^^42^ The economic development of a province, as represented by GDP per capita affects residents’ access to healthcare and public services, thereby shaping their overall health status. It also reflects the region’s capacity for disaster preparedness and recovery, which can mitigate the impact of the TC. Vegetation cover measured by NDVI has direct and indirect effects on health. It can reduce stress and improve air quality and serve as a natural buffer against high winds and storm surge associated with TCs. By controlling for these 2 variables, we can better ascertain the public health consequences from the cyclones, allowing better estimation of the real effect.

### Statistical analysis

The basic analytic model of this study is shown in eqn (1):


(1) $$\begin{array}{c} {\text{Score}}_{\mathrm{ijt}}=\alpha_{1}T_{i,t\_0}+\alpha_{2}T_{i,t\_7}+\alpha_{3}T_{i,t\_30}+\alpha_{4}T_{i,t\_90}+\alpha_{5}T_{i,t\_365}+\alpha_{6}T_{i,t\_1460}+\alpha_{7}T_{i,t\_1460\_\text{above}}+\delta {X^{\prime }}_{\mathrm{ijt}}\\ +E_{2}+E_{3}+\chi_{j}+\lambda_{i}+\mu_{t}+\eta_{t}+\prime_{\mathrm{ijt}} \end{array}$$

$\mathrm{Scor}e_{\mathrm{ijt}}$ represented the cognitive function test scores for individual i in province j at date t. t was for the interview date. $T_{i,t\_0}$ measured an individual’s exposure to TCs on the date t, ie, same-day exposure. $T_{i,t\_7}$ measured an individual’s exposure to TCs between 1 and 7 days prior to date t, ie, exposure within 1 week. $T_{i,t\_30}$ measured an individual’s exposure to TCs between one week and one month before date t, ie, exposure within one month. $T_{i,t\_90}$ measured an individual’s exposure to TCs between one and 3 months prior to date t. $T_{i,t\_365}$ measured an individual’s exposure to TCs between 3 month and one year prior to date t. $T_{i,t\_1460}$ measured an individual’s exposure to TCs between one and 4 years prior to date t.$T_{i,t\_1460\_\mathrm{above}}$ measured an individual’s exposure to TCs that occurred more than 4 years prior to date t. $X_{\mathrm{ijt}}$ denoted a set of covariates. $E_{2}$ and $E_{3}$ indicated whether individuals were exposed to TCs during any 2 or all 3 periods considered, respectively, to account for the potential effect of individuals experiencing multiple TCs over time on the estimates.^38^  $\lambda_{i}$ denoted individual fixed effects. $\chi_{j}$ represented the provincial level fixed effect, which could not be absorbed by the individual fixed effect because some individuals did not reside in the same province across waves. $\mu_{t}$ denoted year fixed effects. $\eta_{t}$ indicated interview month fixed effects to account for potential seasonal trends that may exist in TCs and cognitive tests. $\prime_{\mathrm{ijt}}$ was the error term, and the standard errors were clustered at the provincial level.

To confirm the robustness of the findings, we conducted 3 robustness tests. First, our estimates were based on the assumptions that the occurrence of TCs was completely random and unpredictable for individuals, and that some time-varying, unobserved factors would not affect the estimates. To test the validity of this hypothesis, we designed a falsification test to examine whether TCs occurring after the interview date affected cognitive test scores. Second, to ensure that potential migration that may have occurred during the time period does not affect study results, we constructed and re-estimated a dataset that included only the samples that did not migrate during the 3 waves of the survey. Third, we also estimated the average effect of TC exposure over the sample period to corroborate the robustness of our findings.

To deepen our knowledge of how TCs might contribute to cognitive decline in older adults, we explored potential indirect mechanisms. The causal steps approach may introduce bias to the estimates due to the endogeneity of the mediator variable itself^43^ and may fail to obtain consistent estimates where the mediator variable and the outcome variable may be mutually causal.^44^ Therefore, we used Dell’s mechanism test framework^45^ to examine the existence of potential indirect mechanisms in this study. This framework focuses on the causal effect of the independent variable on the outcome variable and the mediator variable. If the causal effect of the mediator variable on the outcome variable is theoretically straightforward and has been confirmed by numerous studies, it is not necessary to use formal techniques of causal inference to study this effect. This framework has been widely recognized and used.^46^

In this study, the relationships between cognitive function and 3 types of mechanisms (ie, depression, hypertension, and social isolation) are conceptually sound and widely supported by empirical evidence.^27–29^ Therefore, we used eqn (2) that focuses on the estimation of the effects of TC exposure on 3 types of underlying mechanisms, whereas the


(2) $$\begin{array}{c} {\text{Mechanism}}_{\mathrm{ijt}}=\alpha_{1}T_{i,t\_0}+\alpha_{2}T_{i,t\_7}+\alpha_{3}T_{i,t\_30}+\alpha_{4}T_{i,t\_90}+\alpha_{5}T_{i,t\_365}+\alpha_{6}T_{i,t\_1460}+\alpha_{7}T_{i,t\_1460\_\text{above}}+\delta {X^{\prime }}_{\mathrm{ijt}}\\ +E_{2}+E_{3}+\chi_{j}+\lambda_{i}+\mu_{t}+\eta_{t}+\prime_{\mathrm{ijt}} \end{array}$$

${\text{Mechanism}}_{\mathrm{ijt}}$ in eqn (2) denoted each of the potential underlying mechanisms, and the rest is the same as in eqn (1).

All statistical analyses for this study were performed in Stata 18, StataCorp. LLC, 2023, College Station, TX, USA. *p* < .05 was considered statistically significant.

## Results

Table 1 shows the descriptive statistics of the sample by wave. As shown in Table 1, our sample contained 7212 observations (1444 in 2017, 2406 in 2020, and 3362 in 2022). The average age of the 2017 sample was 72.13 years (*SD* = 8.47), slightly more than half were women, about 60% of the sample was married, about half lived in rural areas, and more than a quarter lived alone and worked. Across waves, more than 90% of the sample was Buddhist, and more than 40% had hypertension.

**Table 1.** Descriptive statistics of participants.

Variables	2017 Wave (*N* = 1444)	2020 Wave (*N* = 2406)	2022 Wave (*N* = 3362)
	Mean (*SD*) or %	Mean (*SD*) or %	Mean (*SD*) or %
Age	72.13 (8.47)	73.63 (9.03)	72.34 (8.79)
Male	45.57%	41.31%	38.07%
Married	60.25%	53.33%	52.47%
Employed	26.80%	22.61%	32.69%
Years of education	6.21 (2.44)	6.25 (2.64)	6.65 (2.73)·
Religion			
No religion	0.00%	0.08%	0.03%
Buddhist	91.20%	91.52%	92.89%
Christian	0.28%	0.42%	0.48%
Muslims	8.52%	7.94%	6.31%
Other religions	0.00%	0.04%	0.30%
Living alone	27.84%	13.90%	12.64%
Urban resident	51.04%	49.46%	57.11%
Engaging in smoking	12.33%	9.35%	8.89%
Engaging in drinking	14.68%	9.89%	14.07%
Engaging in exercise	57.13%	49.71%	51.28%
Hospital-based outpatient care	50.28%	50.71%	44.56%
Community health center-based outpatient care	37.88%	21.20%	19.16%
Clinic-based outpatient care	17.80%	9.77%	6.40%
Logarithm of family assets	12.75 (1.63)	6.75 (6.35)	4.12 (5.94)
Logarithm of GDP per capita	11.88 (0.43)	11.91 (0.51)	11.99 (0.54)
Vegetation cover (NVDI)	0.65 (0.09)	0.59 (0.09)	0.62 (0.01)

Data are presented as mean (*SD*) for continuous variables and no. (%) for categorical variables.

Abbreviations: GDP, gross domestic product; NVDI, normalized difference vegetation index.

### The effect of TC exposure on cognitive function in older adults

As shown in Panel A of Table 2, exposure to TCs had no significant effects on memory in either the short term or the long term. Exposure to TCs was found to have consistent negative effects on calculation abilities in both the short term and the long term (Table 2, Panel B). Specifically, this negative effect persisted through 4 years. However, this negative effect was not significant more than 4 years after exposure.

**Table 2.** The effect of TC exposure on cognitive function in older adults.

Panel A	Memory								
Exposure	Total			Immediate recall test			Delayed recall test		
	*B*	*SE*	*p*	*B*	*SE*	*p*	*B*	*SE*	*p*
Same-day	0.918	1.615	.580	1.063	1.176	.384	−0.300	1.012	.772
1 day–1 week	2.018	1.454	.190	0.805	0.687	.264	1.114	0.806	.192
1 week–1 month	1.695	1.430	.259	0.715	0.649	.292	0.888	0.840	.311
1 month–3 months	1.869	1.532	.246	0.906	0.731	.239	0.834	0.856	.349
3 months–1 year	0.408	1.043	.702	0.070	0.512	.894	0.346	0.540	.534
1 year–4 years	1.475	1.036	.180	0.453	0.523	.404	0.998	0.531	.085
>4 years	−0.150	1.267	.907	−0.178	0.561	.757	−0.127	0.776	.873
*N*	5363			5525			5373		
*R*-squared	0.609			0.600			0.589		

Panel B	Calculation		
Exposure	*B*	*SE*	*p*
Same-day	−2.671	0.565	.000
1 day–1 week	−1.289	0.365	.004
1 week–1 month	−1.538	0.498	.009
1 month–3 months	−1.277	0.565	.043
3 months–1 year	−1.231	0.213	.000
1 year–4 years	−1.427	0.467	.010
>4 years	−1.411	1.118	.230
*N*	3442		
*R*-squared	0.671		

Panel C	Time orientation		
Exposure	*B*	*SE*	*p*
Same-day	−0.205	0.567	.725
1 day–1 week	0.052	0.241	.832
1 week–1 month	−0.015	0.114	.896
1 month–3 months	−0.050	0.150	.746
3 months–1 year	−0.299	0.061	.000
1 year–4 years	0.019	0.119	.879
>4 years	0.022	0.173	.899
*N*	4792		
*R*-squared	0.642		

All models were set according to eqn (1).

Abbreviations: *B*, coefficient; *N*, observations; TC, tropical cyclone.

For time orientation (Table 2, Panel C), individuals only experienced a significant decrease in time orientation test scores from 3 months to one year after TC exposure, while the effects of TCs at all other time points were not significant.

We further validated the above results through 3 robustness tests. The results of the falsification test indicated that TCs occurring after the interview date did not affect cognitive test scores, which largely eliminated concerns about potential omitted variables (Supplementary Table 4). Furthermore, the re-estimation results without the immigrant group are consistent with the results of the main analysis (Supplementary Table 5). The results of the estimation of the average effect of TC exposure during the sample period also support our findings (Supplementary Table 6).

### Potential indirect mechanisms of TCs affecting cognition in older adults

All 3 factors have been widely recognized as risk factors to cognitive function,^27–29^ implying that TC exposure can negatively impact cognitive function through these potential indirect pathways. As shown in Panel A of Table 3, on the day of exposure, exposed individuals had a significantly higher risk of developing depression compared to unexposed individuals. This negative effect persisted 4 years after exposure.

**Table 3.** Potential indirect mechanism test results.

Panel A	Having depression		
Exposure	*B*	*SE*	*p*
Same-day	0.263	0.113	.038
1 day–1 week	0.101	0.038	.021
1 week–1 month	0.089	0.031	.014
1 month–3 months	0.101	0.044	.042
3 months–1 year	0.101	0.021	.000
1 year–4 years	0.107	0.025	.001
>4 years	0.141	0.041	.005
*N*	7205		
*R*-squared	0.552		

Panel B	Having hypertension		
Exposure	*B*	*SE*	*p*
Same-day	0.054	0.039	.192
1 day–1 week	0.049	0.047	.319
1 week–1 month	0.029	0.039	.474
1 month–3 months	0.024	0.054	.662
3 months–1 year	−0.014	0.024	.584
1 year–4 years	0.010	0.030	.758
>4 years	0.062	0.058	.302
*N*	7008		
*R*-squared	0.856		

Panel C	Severity of hypertension		
Exposure	*B*	*SE*	*p*
Same-day	——	——	——
1 day–1 week	0.058	0.034	.117
1 week–1 month	0.016	0.046	.738
1 month–3 months	−0.056	0.048	.262
3 months–1 year	0.096	0.051	.082
1 year–4 years	0.349	0.071	.000
>4 years	0.351	0.058	.000
*N*	2807		
*R*-squared	0.779		

Panel D	Social isolation		
Exposure	*B*	*SE*	*p*
Same-day	−0.185	0.178	.320
1 day–1 week	0.043	0.052	.431
1 week–1 month	0.002	0.055	.978
1 month–3 months	0.024	0.070	.735
3 months–1 year	0.151	0.055	.018
1 year–4 years	0.270	0.089	.010
>4 years	——	——	——
*N*	2406		
*R*-squared	0.353		

Same-day exposure omitted because of collinearity in the estimation of severity of hypertension. Exposure over 4 years omitted because of collinearity in the estimate of social isolation. All models follow the setup in equation (2), except for the estimation of social isolation, which is only available for the 2020 wave. For social isolation, the model includes the same covariates as in equation (2) but is adjusted to include only province-level fixed effects, interview month fixed effects, and standard errors clustered at the provincial level.

Abbreviations: *B*, coefficient; *N*, observations.

Regarding hypertension, exposure to TCs did not significantly affect the risk of developing hypertension in exposed individuals at all time point (Table 3, Panel B). However, as shown in Panel C of Table 3, it did significantly raise the risk of continued deterioration in individuals with preexisting hypertension within 1 to 4 years of exposure, and such impact continued after 4 years of exposure.

In terms of social isolation (Table 3, Panel D), TCs were associated with a significantly higher probability of individuals not participating in social activities between 3 months and one year after exposure. This probability increased over time, with individuals significantly more likely to refrain from social activities between 1 and 4 years after exposure.

## Discussion

To the best of our knowledge, our study is the first of its kind that examined the impact of TC exposure on cognitive function of older adults, extending the existing literature that primarily examined the effects of TCs on psychological stress and physical health.^1^^,^^2^ Additionally, our findings elucidated the adverse effects of TCs on certain cognitive domains using a relatively large longitudinal sample and distinguished the nuanced effects of TC exposure over different time frames (immediate, short-term, and long-term).

Our primary finding is that TCs had a significant impact on the calculation domain. Calculation tasks typically require sustained mental effort, concentration, and cognitive flexibility. Therefore, calculation represents a higher cognitive demand than tasks that require recalling simple information or being oriented to time. Because calculation engages multiple cognitive domains, it is often more precise to detect cognitive decline than tasks involving memory or time orientation. Difficulty with calculations can indicate impairments in executive function, processing speed, working memory, or attention—all of which are crucial for daily functioning and can signal various forms of cognitive impairment or dementia. We suspect that the lack of a persistent significant effect of TCs on memory or time orientation may be because the 2 less complex domains are more susceptible to fluctuation due to noncognitive conditions (eg, lack of sleep, not paying attention, no interest). Nonetheless, we believe more research is needed to examine the differential effects of TCs on various cognitive domains.

Previous studies have established that natural disasters can impact overall cognitive function and certain cognitive domains. For example, research on the 2011 Great East Japan Earthquake and Tsunami identified a decline in global cognition among affected older adults,^47^ whereas a study on the 2010 earthquake in Christchurch, New Zealand found specific negative effects on attention.^48^ Our results extended the evidence to the domain of calculation. Additionally, we identified that the impact on calculation began on the day of exposure and persisted over time, suggesting long-term effects on cognition.

The other primary finding of this study is that an increased likelihood of depression, the exacerbation of hypertension, and social isolation may serve as potential indirect mechanisms underlying the impact of TCs on calculation. These findings are consistent with previous cross-sectional studies, which suggested that TCs may increase the risk of depression, worsen the severity of hypertension in existing patients, and lead to social isolation.^18–23^^,^^25^^,^^26^ Using a longitudinal study design with relatively large samples and the fixed effects model, we found that exposure to TCs significantly increased the risk of depression, worsened outcomes for individuals with preexisting hypertension, and heightened the risk of social isolation. However, exposure to TCs did not affect the onset of hypertension, which aligned with the findings of some existing studies.^5^ Furthermore, depression, the exacerbation of hypertension, and social isolation have been widely documented in numerous studies contributing to cognitive decline,^27–29^ supporting the findings of this study on the potential indirect mechanisms through which TCs might affect cognitive function.

The estimates of the impact of TCs appeared to be unbiased and robust, as TCs were pervasive exogenous shocks, providing the basis for obtaining relatively clean estimates in this study. However, several limitations should be noted. First, the measures of individual cognitive function in the HART survey did not include a global measure of cognitive function, restricting this study to 3 distinct cognitive domains. Second, due to the limitations of the HART data, records on individual social activities were only available in the 2020 wave. Furthermore, we explored only certain potential mechanisms between exposure to TCs and cognitive function decline. For example, PTSD-related variables, which can be critical for post-disaster cognitive function, were not available. Third, 4565 observations in the age 60 and older sample of this study were excluded due to significant missingness. The excluded participants appeared to be different in some demographic characteristics from the included sample (see Supplementary Table 7), which may introduce selection bias. Finally, our study design did not allow for an examination of the cumulative effects of repeated TC exposures, an analysis that would be highly valuable for future risk stratification efforts.

The health consequences of TCs identified in this study have important practice and policy implications. Practically, it is crucial to assess all health impacts of TCs, including cognitive function, and to incorporate trauma-informed care in standard practices. Proactive measures, such as preparing for all weather seasons, raising public awareness about the impact of TCs, and enhancing disaster preparedness, are essential. Additionally, further research is needed to monitor the threats of TCs due to climate change and to examine their health outcomes on older adults.

Policy-wise, a comprehensive approach that includes infrastructure development, environmental sustainability, social inclusivity, and health promotion is needed to mitigate cognitive risks such as depression, hypertension, and social isolation.^49^ For example, Bangkok’s “Resilient City” program, launched in 2017, promotes green spaces, social engagement, and access to healthcare,^50^ serving as a model for supporting older adults in coping with natural disasters both short- and long-term. Adaptation strategies like these have broader relevance across Thailand, considering its varied geography and socioeconomic conditions.

## Conclusion

As the first study of its kind that examined the effects of TCs on cognitive function in older adults, this longitudinal study demonstrated the long-term detrimental effects of TC on calculation cognitive function in older adults and identified 3 potential indirect mechanisms, such as depression, exacerbation of hypertension, and social isolation. Rather than focusing on a single TC, this study integrated an assessment of the impacts of multiple cyclone disasters that occurred during the sample period. The findings of this study emphasized the importance of practice and policy interventions to address the impact of TCs on cognitive function among older adults in Thailand, a country facing multiple climate-related threats and a growing aging population.

## Supplementary Material

glaf169_Supplementary_Data

## Data Availability

Data supporting the results of this study are available on request from the Health, Aging, and Retirement in Thailand (HART) project (https://hart.nida.ac.th/).
