# Supplementary material for: The effect of tropical cyclone on cognitive function in older adults: a longitudinal study from Thailand
Source: J Gerontol A Biol Sci Med Sci. 2025 Jul 29;80(9):glaf169. doi: 10.1093/gerona/glaf169 (PMC12449064; doi:10.1093/gerona/glaf169)
Supplement: glaf169_Supplementary_Data [file glaf169_supplementary_data.docx]

**The effect of tropical cyclone on cognitive function in older adults: a longitudinal study from Thailand**

**Supplementary materials**

[**Supplementary Table 1. Detailed tropical storm distribution for the 13 provinces in the 2017-2022 HART sample** 3](#_Toc193059823)

[**Supplementary Table 2. Descriptive statistics of cognitive test scores** 4](#_Toc193059824)

[**Supplementary Table 3. Descriptive statistics of potential mechanism variables** 4](#_Toc193059825)

[**Supplementary Table 4. falsification test** 6](#_Toc193059826)

[**Supplementary Table 5. Re-estimation of the nonimmigrant sample** 7](#_Toc193059827)

[**Supplementary Table 6. The average effect of exposure to TCs** 8](#_Toc193059828)

[**Supplementary Table 7. Comparison analysis of characteristics of the included and excluded observations** 9](#_Toc193059829)

**Supplementary Table 1. Detailed tropical storm distribution for the 13 provinces in the 2017-2022 HART sample**

| **No.** | **Start Year** | **Start Month** | **Start Day** | **End Year** | **End Month** | **End Day** | **Location** |
| --- | --- | --- | --- | --- | --- | --- | --- |
| 1 | 2017 | 9 | 17 | 2017 | 9 | 17 | Phetchabun; Uttaradit |
| 2 | 2020 | 4 | 29 | 2020 | 5 | 9 | Bangkok; Nonthaburi; Pathum Thani; Chanthaburi; Samut Prakan; Surin; Khon Kaen; Chiang Mai; Uttaradit; Krabi; Songkhla |
| 3 | 2020 | 10 | 9 | 2020 | 10 | 31 | Bangkok; Nonthaburi; Pathum Thani; Chanthaburi; Samut Prakan; Phetchabun; Songkhla |
| 4 | 2020 | 9 | 18 | 2020 | 9 | 21 | Chanthaburi; Chiang Mai; Uttaradit; Phetchabun |
| 5 | 2021 | 3 | 22 | 2021 | 3 | 23 | Surin; Chanthaburi; Khon Kaen; Chiang Mai; Uttaradit; Phetchabun |
| 6 | 2022 | 5 | 5 | 2022 | 5 | 9 | Chiang Mai |

Notes: All information is derived from the EM-DAT Disaster Database and reconfirmed using the ASEAN (Association of Southeast Asian Nations) Disaster Information Network (ADINet). ADINet is operated by the ASEAN Disaster Management and Humanitarian Coordination Center (AHA Centre), which provides detailed information on when, where, and to what extent natural disasters (e.g. floods, typhoons, earthquakes, etc.) occur.

**Supplementary Table 2. Descriptive statistics of cognitive test scores**

|  | **Non-exposed group** | **Exposure group** | **Total** |
| --- | --- | --- | --- |
|  | **Mean (SD)** | **Mean (SD)** | **Mean (SD)** |
| Memory Total Test Score | 5.326 (3.355) | 7.533 (3.727) | 6.832 (3.756) |
| Immediate Recall Test Score | 3.123 (1.789) | 4.168 (1.905) | 3.845 (1.931) |
| Delayed Recall Test Score | 2.201 (1.864) | 3.303 (2.052) | 2.954 (2.059) |
|  |  |  |  |
| Calculated Test Score | 2.090 (1.780) | 2.590 (1.846) | 2.423 (1.839) |
| Time Orientation Test Score | 3.350 (1.064) | 3.742 (0.738) | 3.611 (0.879) |

Notes: SD = Standard deviation.

**Supplementary Table 3. Descriptive statistics of potential mechanism variables**

|  | **Total**  **n (%)** | **Obs**  **(Total)** | **Non-exposed group**  **n (%)** | **Obs**  **(Non-exposed group)** | **Exposure group**  **n (%)** | **Obs**  **(Exposure group)** |
| --- | --- | --- | --- | --- | --- | --- |
| Having depression  (1 for yes, 0 for no) | 470 (6.52) | 7205 | 160 (8.23) | 1945 | 310 (5.89) | 5260 |
| Having hypertension  (1 for yes, 0 for no)  Severity of hypertension  (1 for more severe, 0 for less severe) | 3444(49.14)  878(31.28) | 7008    2807 | 939 (48.45)  157 (51.99) | 1938  302 | 2505 (49.41)  721 (28.78) | 5070  2505 |
| Social isolation  (1 for not having social activities, 0 for not) | 1345(55.90) | 2406 | 175 (75.76) | 231 | 1170 (53.79) | 2175 |

Notes: Severity of hypertension is only available in waves 2020 and 2022. Social isolation is only available in the 2020 wave.

**Supplementary Table 4. falsification test**

| **Memory Total Test Score** | **B** | **SE** | ***p*** |
| --- | --- | --- | --- |
| TCs that occurred after the interview date | -0.013 | 0.975 | 0.989 |
| N | 5363 | | |
| R-squared | 0.596 | | |
| **Calculated Test Score** | **B** | **SE** | ***p*** |
| TCs that occurred after the interview date | -0.156 | 0.669 | 0.819 |
| N | 3442 | | |
| R-squared | 0.662 | | |
| **Time Orientation Test Score** | **B** | **SE** | ***p*** |
| TCs that occurred after the interview date | -0.019 | 0.144 | 0.895 |
| N | 4792 | | |
| R-squared | 0.637 | | |

Notes: B = Coefficient. N = Observations. SE = Standard error. All models control for the same covariates and fixed effects in equation (1).

**Supplementary Table 5. Re-estimation of the nonimmigrant sample**

| **Memory Total Test Score** | **B** | **SE** | ***p*** |
| --- | --- | --- | --- |
| Same-day | 0.905 | 1.209 | 0.468 |
| 1 day – 1 week | 2.010 | 1.091 | 0.090 |
| 1 week – 1 month | 1.683 | 1.069 | 0.141 |
| 1 month – 3 months | 1.843 | 1.142 | 0.132 |
| 3 months – 1 year | 0.406 | 0.784 | 0.614 |
| 1 year – 4 years | 1.482 | 0.784 | 0.083 |
| > 4 years | -0.151 | 0.950 | 0.876 |
| N | 5360 | | |
| R-squared | 0.609 | | |
| **Calculated Test Score** | **B** | **SE** | ***p*** |
| Same-day | -2.629 | 0.388 | 0.000 |
| 1 day – 1 week | -1.283 | 0.267 | 0.000 |
| 1 week – 1 month | -1.527 | 0.362 | 0.001 |
| 1 month – 3 months | -1.246 | 0.400 | 0.009 |
| 3 months – 1 year | -1.228 | 0.156 | 0.000 |
| 1 year – 4 years | -1.445 | 0.341 | 0.001 |
| > 4 years | -1.413 | 0.818 | 0.110 |
| N | 3439 | | |
| R-squared | 0.671 | | |
| **Time Orientation Test Score** | **B** | **SE** | ***p*** |
| Same-day | -0.205 | 0.423 | 0.637 |
| 1 day – 1 week | 0.052 | 0.180 | 0.776 |
| 1 week – 1 month | -0.015 | 0.085 | 0.860 |
| 1 month – 3 months | -0.050 | 0.112 | 0.663 |
| 3 months – 1 year | -0.299 | 0.046 | 0.000 |
| 1 year – 4 years | 0.019 | 0.089 | 0.838 |
| > 4 years | 0.022 | 0.129 | 0.865 |
| N | 4789 | | |
| R-squared | 0.642 | | |

Notes: B = Coefficient. N = Observations. SE = Standard error. All models were set according to equation (1).

**Supplementary Table 6. The average effect of exposure to TCs**

| **Memory Total Test Score** | **B** | **SE** | ***p*** |
| --- | --- | --- | --- |
| Average effect | 0.976 | 0.921 | 0.310 |
| N | 5363 | | |
| R-squared | 0.597 | | |
| **Calculated Test Score** | **B** | **SE** | ***p*** |
| Average effect | -1.112 | 0.186 | 0.000 |
| N | 3442 | | |
| R-squared | 0.665 | | |
| **Time Orientation Test Score** | **B** | **SE** | ***p*** |
| Average effect | -0.150 | 0.077 | 0.077 |
| N | 4792 | | |
| R-squared | 0.636 | | |

Notes: B = Coefficient. N = Observations. SE = Standard error. All models control for the same covariates and fixed effects in equation (1).

**Supplementary Table 7. Comparison analysis of characteristics of the included and excluded observations**

| **Variables** | **Obs**  **(exclusion group)** | **Mean**  **(exclusion group)** | **Obs**  **(inclusion group)** | **Mean**  **(inclusion group)** | **mean-diff** | **t** |
| --- | --- | --- | --- | --- | --- | --- |
| Age | 1596 | 75.071 | 7212 | 72.732 | 2.340*** | 9.468 |
| Male | 1676 | 0.448 | 7212 | 0.407 | 0.042*** | 3.112 |
| Married | 1662 | 0.507 | 7212 | 0.543 | -0.036*** | -2.647 |
| Employed | 1225 | 0.221 | 7212 | 0.281 | -0.060*** | -4.382 |
| Years of education | 1603 | 6.379 | 7212 | 6.427 | -0.048 | -0.622 |
| Urban residents | 4163 | 0.572 | 7212 | 0.533 | 0.039*** | 4.004 |
| Engaging in smoking | 1675 | 0.108 | 7212 | 0.097 | 0.011 | 1.321 |
| Engaging in drinking | 1673 | 0.104 | 7212 | 0.128 | -0.024*** | -2.687 |
| Engaging in exercise | 1664 | 0.511 | 7212 | 0.519 | -0.008 | -0.622 |
| Logarithm of family assets | 1103 | 4.622 | 7212 | 6.726 | -2.104*** | -10.215 |
| Logarithm of GDP per capita | 4565 | 12.083 | 7212 | 11.945 | 0.139*** | 12.970 |
| Vegetation Cover (NVDI) | 4565 | 0.610 | 7212 | 0.614 | -0.004** | -2.415 |

Notes: GDP = Gross domestic product. NVDI = Normalized difference vegetation index.
